# Developing high-affinity, oxygen-insensitive [NiFe]-hydrogenases as biocatalysts for energy conversion

**DOI:** 10.1042/BST20230120

**Published:** 2023-09-25

**Authors:** Chris Greening, Ashleigh Kropp, Kylie Vincent, Rhys Grinter

**Affiliations:** 1 Department of Microbiology, Biomedicine Discovery Institute, Monash University, Clayton, VIC 3800, Australia; 2 Securing Antarctica's Environmental Future, Monash University, Clayton, VIC 3800, Australia; 3 Centre to Impact AMR, Monash University, Clayton, VIC 3800, Australia; 4 ARC Research Hub for Carbon Utilisation and Recycling, Monash University, Clayton, VIC 3800, Australia; 5 Department of Chemistry, University of Oxford, Inorganic Chemistry Laboratory, Oxford OX1 3QR, U.K.; 6 Centre for Electron Microscopy of Membrane Proteins, Monash Institute of Pharmaceutical Sciences, Parkville, Victoria 3052, Australia

**Keywords:** [NiFe]-hydrogenase, electrocatalysis, enzyme-based fuel cells, hydrogen

## Abstract

The splitting of hydrogen (H_2_) is an energy-yielding process, which is important for both biological systems and as a means of providing green energy. In biology, this reaction is mediated by enzymes called hydrogenases, which utilise complex nickel and iron cofactors to split H_2_ and transfer the resulting electrons to an electron-acceptor. These [NiFe]-hydrogenases have received considerable attention as catalysts in fuel cells, which utilise H_2_ to produce electrical current. [NiFe]-hydrogenases are a promising alternative to the platinum-based catalysts that currently predominate in fuel cells due to the abundance of nickel and iron, and the resistance of some family members to inhibition by gases, including carbon monoxide, which rapidly poison platinum-based catalysts. However, the majority of characterised [NiFe]-hydrogenases are inhibited by oxygen (O_2_), limiting their activity and stability. We recently reported the isolation and characterisation of the [NiFe]-hydrogenase Huc from *Mycobacterium smegmatis*, which is insensitive to inhibition by O_2_ and has an extremely high affinity, making it capable of oxidising H_2_ in air to below atmospheric concentrations. These properties make Huc a promising candidate for the development of enzyme-based fuel cells (EBFCs), which utilise H_2_ at low concentrations and in impure gas mixtures. In this review, we aim to provide context for the use of Huc for this purpose by discussing the advantages of [NiFe]-hydrogenases as catalysts and their deployment in fuel cells. We also address the challenges associated with using [NiFe]-hydrogenases for this purpose, and how these might be overcome to develop EBFCs that can be deployed at scale.

## Introduction

**Figure 1.**
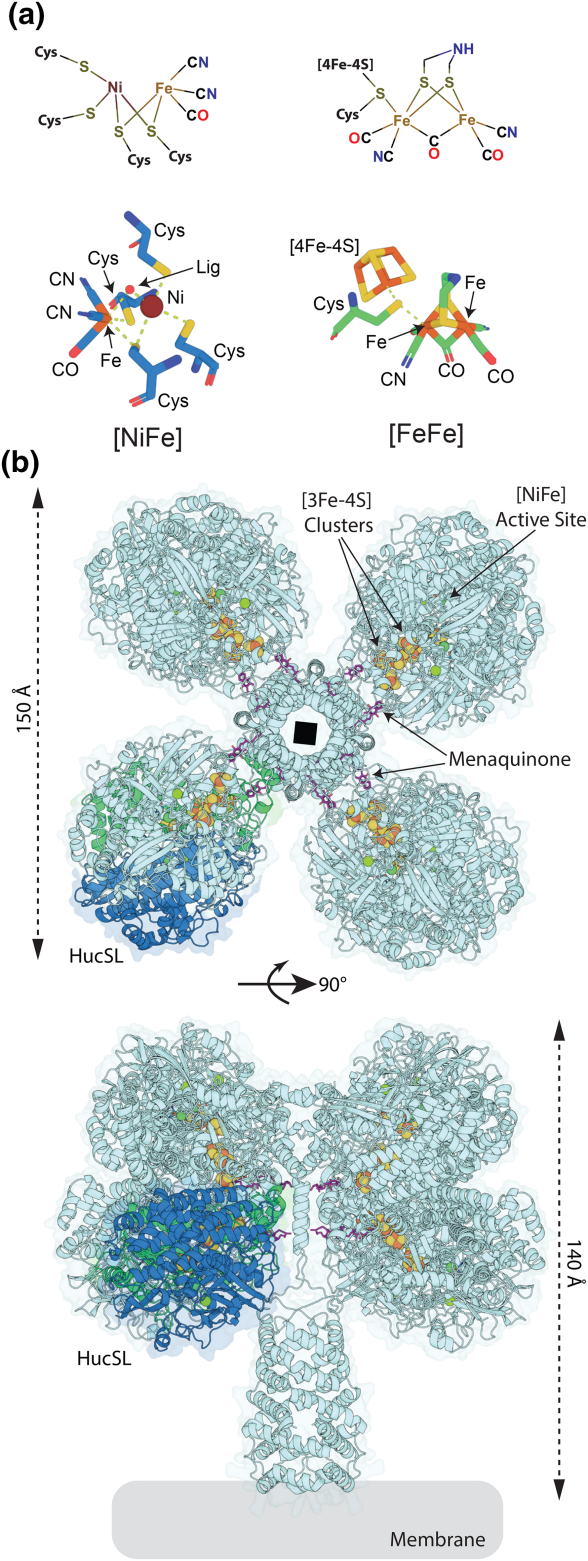
The catalytic structure of [FeFe] and [NiFe]-hydrogenases. (a) The architecture of the catalytic clusters of [NiFe] and [FeFe]-hydrogenases. Lig = H_2_ or OH depending of the state of the catalytic cluster. (**b**) The CryoEM structure of the complex [NiFe]-hydrogenase Huc from *M. smegmatis* (PDB ID = 7UUS). A single catalytic unit consisting of HucS and HucL subunits (HucSL) is indicated, as are the [NiFe]-catalytic cluster, electron transferring [3Fe-4S] clusters, and electron accepting menaquinone.

Hydrogenases are enzymes that perform the simplest chemical reaction: the interconversion of molecular hydrogen (H_2_) with two protons and two electrons (H_2_ ⇔ 2e^−^ + 2H^+^). Despite the simplicity of this reaction, hydrogenases are complicated enzymes, which require specialised metal cofactors for both catalysis and electron transfer. Two distinct classes of hydrogenase have evolved to catalyse this reaction, designated [NiFe] and [FeFe] based on their active site architecture [1]. Though phylogenetically and structurally unrelated, these classes of hydrogenase have commonalities in their active site architecture, including iron coordinated by carbon monoxide and at least one cyanide ligand (Figure 1a) [2–4]. Hydrogenases allow microorganisms to use H_2_ as an energy source by yielding low-potential electrons for respiration and carbon fixation. Other microbes produce H_2_, as a means of disposing of excess reductant during fermentation, as a terminal electron-acceptor, or to maintain redox balance during photosynthesis [5–7]. Additionally, some hydrogenases are capable of bifurcation, coupling the oxidation of H_2_ to the simultaneous reduction in a high and low-potential substrate, or confurcation via the reverse process [8–10].

Hydrogenases have received considerable attention in recent decades for their application as biocatalysts for both biohydrogen production and H_2_ oxidation as an energy source in enzymatic fuel cells [11–19]. [FeFe]-hydrogenases are generally fast-acting enzymes, catalysing the formation and oxidation of molecular hydrogen at rates exceeding 10^3^ s^−1^, which makes them promising enzymes for fermentative or photochemical hydrogen production [20]. However, most are irreversibly inactivated by even traces of oxygen (O_2_), which limits the scope for practical application [21]. Early work on isolated [NiFe]-hydrogenases focussed on enzymes from anaerobes, and like the [FeFe]-hydrogenase, these enzymes were highly sensitive to inhibition by O_2_ [22,23]. However, unlike [FeFe]-hydrogenases, this inhibition is largely reversible via the application of external reductant [24,25]. Later, [NiFe]-hydrogenases isolated from aerobic bacteria were shown to exhibit some tolerance to O_2_, maintaining a considerable fraction of their activity in the presence of O_2_, providing that sufficient H_2_ or reductant were present [26–28]. These ‘O_2_-tolerant’ hydrogenases have shown considerable scope as H_2_ oxidising catalysts for fuel cells and sensors, leading to the development of devices producing useful amounts of electrical current [13,14,16]. However, O_2_ sensitivity remains a problem, limiting the stability of hydrogenase-mediated H_2_ oxidation in enzymatic fuel cells, particularly when operating at high potentials [11,29]. There has also been much progress in developing chemical catalysts that are structurally similar to hydrogenase active sites (biomimetics) [30–32].

Over the past decade, it has been shown that diverse bacteria and archaea use the trace quantities of H_2_ in the atmosphere to support aerobic respiration. We have shown that, through this process, microorganisms gain sufficient energy for mixotrophic growth and survival even when starved of their preferred substrates [33–39]. This ability is mediated by high-affinity [NiFe]-hydrogenases, which can extract and oxidise H_2_ at the levels present in the atmosphere (0.53 parts per million (ppm)/0.41 nM in solution) [40–43]. Recently, we isolated and characterised the [NiFe]-hydrogenase Huc, which mediates atmospheric H_2_ oxidation by the bacterium *Mycobacterium smegmatis* (Figure 1b) [33,40,44]. We demonstrated that Huc has an extremely high affinity for H_2_, allowing it to oxidise H_2_ at well below atmospheric concentrations. Moreover, we showed that Huc is completely insensitive to inhibition by O_2_, a property that allows it to function in ambient air, which contains 20.95% O_2_ [40]. These unique properties make Huc, and other uncharacterised [NiFe]-hydrogenases from atmospheric H_2_ oxidising bacteria, promising candidates for the development of hydrogenase-based electrodes for fuel cells and H_2_ sensors.

In this review, we will highlight the advantages and current progress in the use of [NiFe]-hydrogenases in electrocatalysis, with a focus on their application in fuel cells, as well as the challenges in their development for this purpose and how novel hydrogenases like Huc could help to overcome them.

## Advantages and challenges of hydrogenases as fuel cell catalysts

**Figure 2.**
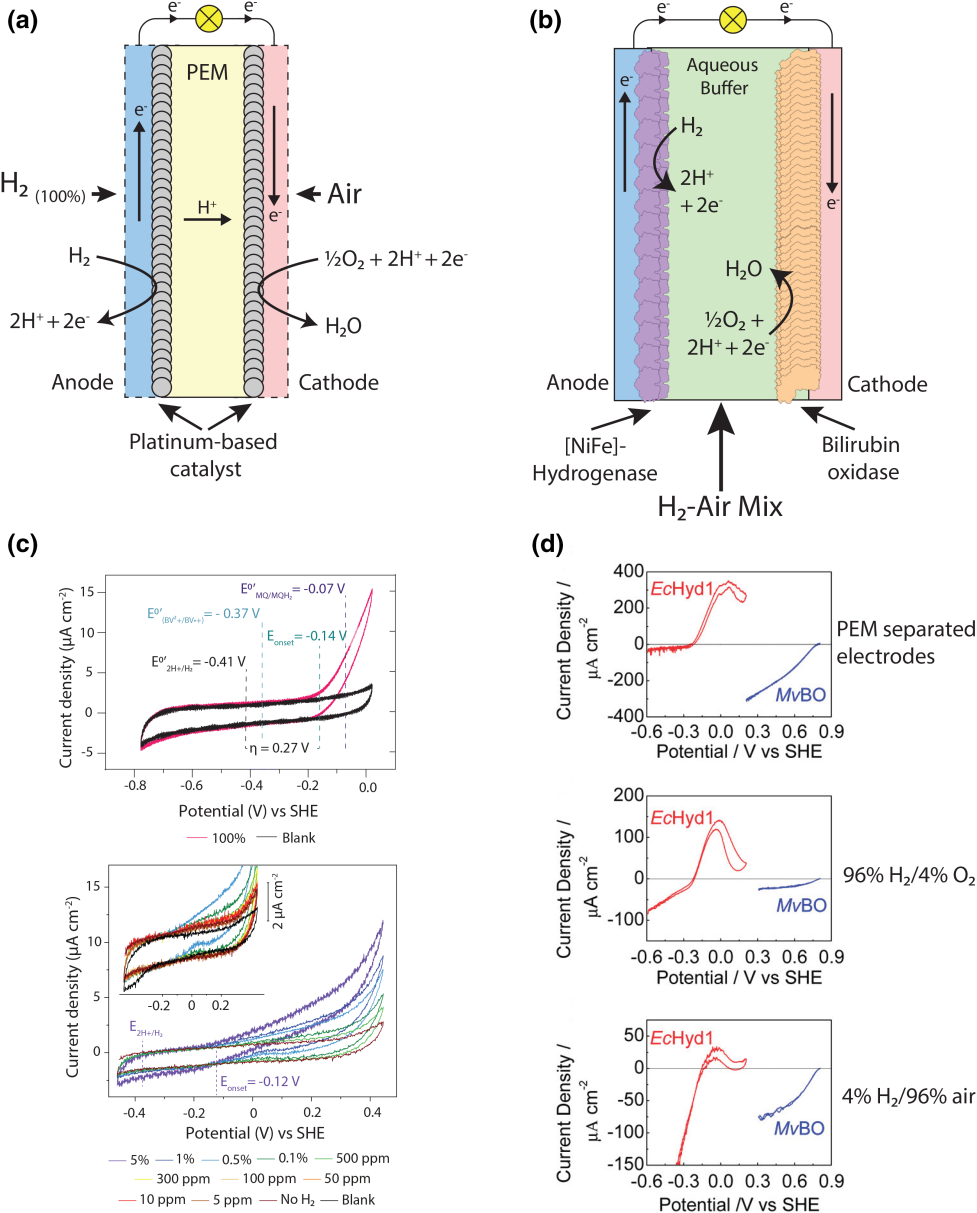
The application of [NiFe]-hydrogenases in fuel cell development. (**a**) A simplified schematic of the general design for a PEMFC. (**b**) A simplified schematic for the general design of a membrane-less [NiFe]-hydrogenase EBFC, similar to those described by Xu and Armstrong [14]. (**c**) Cyclic voltammograms showing the current produced by Huc in the presence of different concentrations of H_2_, adapted from Grinter and Kropp et al. [40] (**d**) Cyclic voltammograms showing the performance of Hyd1 from *E. coli* (*EcHyd1*) and Bilirubin oxidase from *Myrothecium verrucaria* (*MvBO*) in different H_2_ gas mixtures. Oxidative inactivation of *EcHyd1* is observed at potentials of greater than ∼0.0 V vs SHE, adapted with permission from Wait et al. [56].

Proton-exchange membrane fuels cells (PEMFCs) are currently the preeminent fuel cell technology for the conversion of H_2_ to electricity [45]. These fuels cells generally use a platinum-based catalyst at both electrodes, with the anode performing H_2_ oxidation (H_2_ → 2e^−^ + 2H^+^) and the cathode performing O_2_ reduction (½O_2_ + 2e^−^ + 2H^+^ → H_2_O) [46,47]. The reactivity of both electrodes towards O_2_ necessitates their separation using a polymer-based proton-exchange membrane (PEM) to avoid short-circuiting, although fuel crossover remains a problem since membranes are rarely completely impermeable to the gases (Figure 2a) [48,49]. While PEMFCs have been successfully employed to generate electricity for both mobile and static applications, the high cost and limited supply of platinum and proton exchange membranes, have limited their deployment at scale [50].

[NiFe]-hydrogenases as catalysts in enzyme-based fuel cells (EBFCs) offer several advantages over noble metal catalysts employed in PEMFCs. Firstly, H_2_ oxidation by these enzymes is catalysed by nickel and iron, precluding the need for platinum or other precious metals in these fuel cells [1]. This mitigates raw material scarcity for catalyst manufacture and simplifies materials disposal by precluding the need for recycling fuel cells at their end of life for metal recovery. Secondly, the specificity and O_2_ tolerance of some [NiFe]-hydrogenases can mitigate the need for a gas-impermeable membrane separating the fuel cell electrodes (Figure 2b), opening considerable possibilities for miniaturisation [51,52]. Thirdly, the platinum catalysts used by PEMFC are highly sensitive to poisoning by a range of inhibitors, including carbon monoxide (CO) and hydrogen sulphide (H_2_S), meaning that high-purity H_2_ (generally 100%) and air streams are required [53,54]. [NiFe]-hydrogenases can be highly resistant to these inhibitors and so can function using mixed gas streams [55]. Fourthly, high-affinity hydrogenases like Huc from *M. smegmatis* can convert H_2_ into electrical current at low partial pressures that are inaccessible to platinum catalysts (Figure 2c) [40]. These last two points could allow [NiFe]-hydrogenase EBFCs to extract energy from gas streams that are inaccessible to PEMFC, for example, syngas, biogas, flue gas, or in the case of Huc, from the air itself.

As discussed in the next section, significant progress has been made in the development of hydrogenases as electrocatalysts. However, the inactivation of standard hydrogenases in the presence of O_2_ and at high redox potentials has limited their development [11,56]. Huc from *M. smegmatis* is completely insensitive to inhibition by O_2_ and it is not inactivated at high potentials, indicating that it offers promise for overcoming these challenges for EBFCs (Figures 1b, 2c) [40]. However, before the promise of [NiFe]-hydrogenases can be realised, significant work is required to engineer the stability of these enzymes and upscale their production.

## Development of [NiFe]-hydrogenases in EBFCs

The use of [NiFe]-hydrogenases in fuel cells has been investigated since at least 1993, in pioneering work developing hydrogenase electrodes [57]. The first complete hydrogenase-containing fuel cell was reported in 2001, consisting of whole cells of the [NiFe]-hydrogenase-producing bacterium *Desulfovibrio vulgaris* immobilised on the anode, and the O_2_-reducing enzyme bilirubin oxidase on the cathode. This configuration was effective, with the cell operating at 1.0 V producing a current of 0.9 mA, although a gas-impermeable membrane between the electrodes was required to protect the obligately anaerobic *D. vulgaris* cells [58,59]. This voltage is close to the theoretical maximum (open circuit) voltage of 1.23 V, for an H_2_/O_2_ fuel cell with the anode and cathode at 1 bar H_2_ and O_2_, respectively. A fully enzymatic [NiFe]-hydrogenase-containing fuel cell was described only a few years later by Vincent et al. [55]. This fuel cell used O_2_-tolerant [NiFe]-hydrogenase from *Cupriavidus necator* (formerly *Ralstonia eutropha*) and did not employ a membrane to separate the electrodes [60]. While the hydrogenase was partially inhibited by O_2_, the later iteration of this fuel cell, using a [NiFe]-hydrogenase from *Cupriavidus metallidurans* (formerly *Ralstonia metallidurans*), operated in a 3% H_2_ in air mixture producing a max power output of 5.2 µW cm^−2^ at 500 mV, with three cells in series sufficient to power a digital wrist watch for over 24 h [60].

In the subsequent development of membrane-free EBFCs, the O_2_-tolerant [NiFe]-hydrogenase Hyd-1 from *Escherichia coli* was employed. These cells functioned well under high resistive load conditions which lead to operation at low current density and a voltage close to the open circuit voltage, meaning that the hydrogenase anode is exposed only to mildly oxidising potentials. However, it was found that in H_2_-poor mixtures (4% H_2_ in air), the hydrogenase was oxidatively inactivated when these EBFCs operated under low load [56], since this leads to a high operating voltage which imposes a high potential on the hydrogenase electrode (Figure 2d). To mitigate this, Hyd-1-containing fuel cells were operated under a 78% H_2_/22% air mixture, and with the development of porous 3D electrodes, they achieved a power density of 1.67 mW cm^−2^ at a cell voltage of 0.8 V [61]. Two parallel stacks (4 × 4) of cells connected in series provided sufficient current to power five LEDs and a mechanical clock [14]. Alternative strategies have been employed to mitigate the oxygen sensitivity of [NiFe]-hydrogenases in EBFCs. A membrane-less H_2_/O_2_ EBFC was developed containing O_2_-sensitive MBH from *Desulfovibrio vulgaris* Miyazaki F, in which a gas diffusion system was used to provide pure H_2_ to the anode and air to the cathode, protecting the hydrogenase from rapid inactivation by O_2_. The high concentration of H_2_ at the anode allowed this fuel cell to achieve an impressive power density of 6.1 mW cm^−2^ at 0.72 V [18]. EBFCs containing hydrogenases in redox-active hydrogels have also been developed, which act as an O_2_-reducing matrix and Nerst buffer to prevent oxidative inactivation [15,19,62]. A recent study constructed an EBFC containing an anode with [NiFe]-hydrogenases from *D. vulgaris* Miyazaki F embedded in a viologen-modified polymer matrix. This fuel cell produced a maximum power density of 3.6 mW cm^−2^ at 0.7 V [15]. These strategies avoid short-term inactivation of the hydrogenase even when O_2_-sensitive [NiFe]-enzymes are employed. However, their long-term stability was not tested and slow hydrogenase inactivation due to traces of O_2_ reaching the enzyme's active site is likely [15,18,19,62].

## Challenges in the use of [NiFe]-hydrogenases in electrocatalysis

Despite the significant advances in the application of [NiFe]-hydrogenase in EBFCs over the past two decades, a number of challenges need to be addressed before they reach practical application. As discussed below, these challenges include increasing enzyme yield from microbial culture, improving enzyme stability and O_2_ tolerance, and developing lightweight, conductive, porous electrode architectures to facilitate efficient H_2_ mass transfer and high current per electrode volume.

### [NiFe]-hydrogenase production

**Figure 3.**
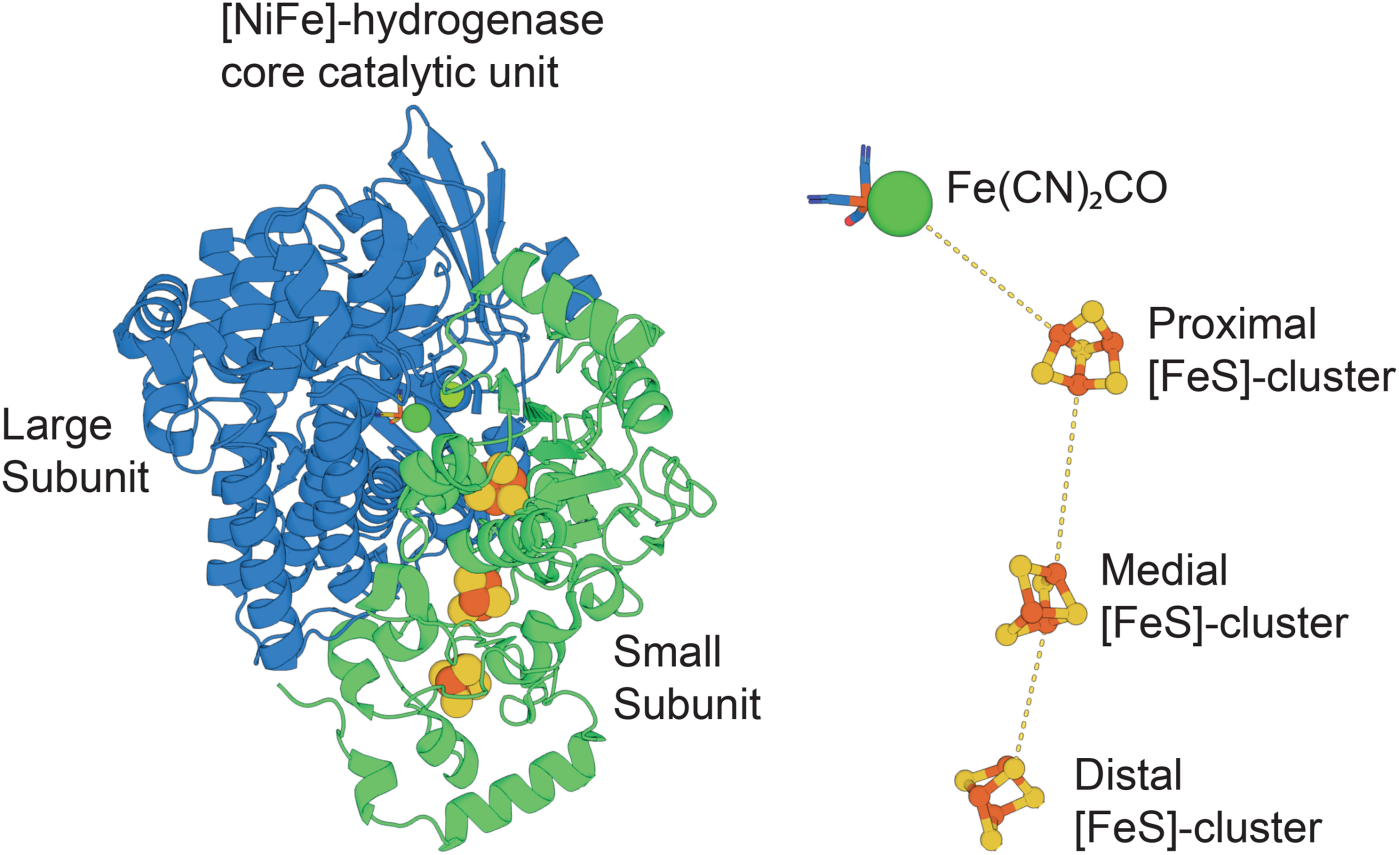
The structure of the [NiFe]-hydrogenase core catalytic complex. The structure of core large and small catalytic subunits of [NiFe]-hydrogenases (left), and the arrangement of catalytic [NiFe] and electron transferring [FeS] cofactors present in the [NiFe]-hydrogenases large and small subunits (right). In this figure, the large and small subunits of Huc are shown for illustrative purposes (PDB ID = 7UUR).

The core catalytic unit of [NiFe]-hydrogenases consists of a ∼90 kDa heterodimer of a large subunit, which contains the catalytic nickel-iron cluster, and a small subunit that contains an electron relay usually composed of three iron-sulfur clusters (Figure 3) [1]. Due to their size and the complexity of their catalytic and redox cofactors, currently [NiFe]-hydrogenases must be synthesised biologically and are produced by purification from microbial cells [40,63]. Unlike [FeFe]-hydrogenases where the catalytic di-iron site can be synthetically matured [64,65], [NiFe]-hydrogenases must currently be enzymatically matured. All characterised [NiFe]-hydrogenases are matured through the combined actions of six dedicated assembly proteins that synthesise the [NiFe] cofactor and in most cases a specific endopeptidase that cleaves the large subunit in the terminal step of the maturation process [66]. Certain hydrogenases, including Huc, also require additional assembly and maturation proteins that are usually encoded by genes associated with those of the enzymatic subunits [63,67,68]. The oxygen sensitivity and anaerboic expression of most characterised [NiFe]-hydrogenases means that their producing microbes often need to be cultured anaerobically [69–71].

Together, these factors contribute to the modest purification yields that have been reported for [NiFe]-hydrogenase, of between 0.025 and 0.15 mg/g wet cell mass, or 0.13 and 8 mg/L of cell culture, produced in shaking flasks [12,40,63,69,70]. The higher of these reported yields were achieved by recombinant expression and/or genetic optimisation of the producing strain, with associated assembly proteins expressed along with the catalytic subunits [63,72]. For example, expression of the oxygen-sensitive hydrogenase Hyd-2 from *E. coli* was improved by an order of magnitude to 0.15 mg/g wet cell mass by deleting a gene encoding a membrane-anchoring subunit of the enzyme and encoding the small subunit on a multicopy plasmid [69,73]. Huc from *M. smegmatis* is natively expressed at low levels and only under nutrient-limited conditions [44]. However, expression levels were significantly improved by the inactivation of the gene encoding glycerol response regulator GylR [74]. This GylR-deficient strain grows slowly in culture media with glycerol as its sole carbon source, likely due to its inability to produce significant quantities of glycerol-metabolising enzymes, with dramatically improved production of Huc. Huc could be purified from these cells with a yield of 0.13 mg/L of culture media, approximately an order of magnitude increase over native cells [40]. This illustrates that an understanding of the genetic regulation of [NiFe]-hydrogenases can be harnessed to improve their expression levels. Efforts to heterologously express the regulatory hydrogenase from *C. necator* in *E. coli* culminated in a yield of 2 mg/g of dry cell weight at very high cell densities (OD_600_ max = 150) using a batch-fed bioreactor [75,76], demonstrating the effectiveness of a combination of heterologous expression and high-density cell culture.

Despite these efforts to improve [NiFe]-hydrogenase production, yields remain below the levels required for the large-scale use of these enzymes in EBFCs. Improvement in yields will require an improved understanding of the role of maturases in their assembly and the regulation of their expression. These data could then be integrated using a systems biology approach to generate synthetic operons where the expression of maturases and structural proteins are tuned to maximise yield. In concert with this, directed evolution could be applied to the producing strains to tune hydrogenase regulation and cellular metabolism to improve [NiFe]-hydrogenase yield, producing bacterial strains compatible with large-scale high-yield production in bioreactor-based systems.

### [NiFe]-hydrogenase sensitivity to O_2_ and high electrical potential

In the presence of O_2_ or under highly oxidising conditions, most [NiFe]-hydrogenases enter reversibly inhibited states typified by oxygenic species, most likely hydroxide, bound at the bridging site between the Fe and Ni ions in the catalytic cluster, with the Ni oxidised from Ni(II) to Ni(III). These two states are classified as either Ni-A (unready) or Ni-B (ready) states based on the difficulty of enzyme reactivation [77–81]. In the presence of O_2_, O_2_-sensitive hydrogenases enter a mixture of Ni-A and Ni-B states and experience some irreversible inactivation, while O_2_-tolerant hydrogenases only form the Ni-B state which can be reductively activated much more quickly [78]. This allows O_2_-tolerant hydrogenases to function in the presence of O_2_ providing that sufficient H_2_ is present [82]. However, both [NiFe]-hydrogenase types can also enter the inhibited Ni-B at high potentials in the absence of O_2_, limiting the potential range under which [NiFe]-hydrogenase anodes can operate in fuel cells [83]. In contrast, the O_2_-insensitive hydrogenase Huc remains fully catalytically active in air at <0.53 ppm H_2_, although structural and spectroscopic analysis has shown that it can form a Ni-B state [40]. Moreover, Huc was not inactivated by potentials more positive than 0.4 V vs the standard hydrogen electrode (SHE) (Figure 2c) [40]. These characteristics indicate it could be used in EBFCs without inactivation by O_2_ or high potentials. An O_2_-insensitive hydrogenase (Hhy) has also been characterised from *C. necator*, though its rates are too low to be applied in fuel cells [84,85].

### [NiFe]-hydrogenase stability in EBFCs

Electronic devices contain components that have a functional lifespan of months, years, or even decades. Moreover, components in these devices that store or utilise electrical charge must undergo multiple cycles of charge and discharge, and operate under current for extended periods. This contrasts with biological life, where organisms regularly replace many of their components at both a cellular and molecular levels [86]. This creates an apparent paradox for the use of enzymes in electronic devices, as they need to remain active for significantly longer periods than they would in their host organisms. To overcome this for their use in EBFCs, significant work will likely be required to engineer [NiFe]-hydrogenases to achieve the stability required for mainstream applications. To date, limited effort has been made to engineer [NiFe]-hydrogenases with improved stability, with enzymes tested in EBFCs being identical or very similar to those of the producing microbe. Despite this, a number of [NiFe]-hydrogenases in experimental fuel cells have maintained activity for up to 24 h [11,16,51,87]. In their study utilising an H_2_/O_2_ EBFC with membrane-bound hydrogenase from *Aquifex aeolicus* as the anode catalyst to power a wireless device, Monslave et al. [16] reported at least 7 h of continuous operation. In addition, an H_2_/O_2_ EBFC containing Hyd-1 from *E. coli* at the anode was used to power 5 LEDs and a small mechanical clock, reported 100% LED intensity after 8 h [14]. One of the major factors in the decline of [NiFe]-hydrogenase fuel cells is likely to be slow inactivation by O_2_, as steps taken to protect the enzyme are unlikely to achieve 100% fidelity [14,88]. The use of O_2_-insensitive enzymes like Huc has the potential to mitigate this problem improving the longevity of [NiFe]-hydrogenases in EBFCs. Huc is also remarkably resistant to both heating and freezing, though its stability within an EBFC remains to be evaluated [40]. The immobilisation of [NiFe]-hydrogenases or their encapsulation in electrode materials have also been shown to improve their stability [14,89–91]. These kinds of material design approaches could be combined with directed evolution, structure-based design, and chemical modification to develop the robust and O_2_-insensitive [NiFe]-hydrogenase-based electrodes that will be required for EBFCs.

### Efficient coupling of [NiFe]-hydrogenases to fuel cell electrodes

**Figure 4.**
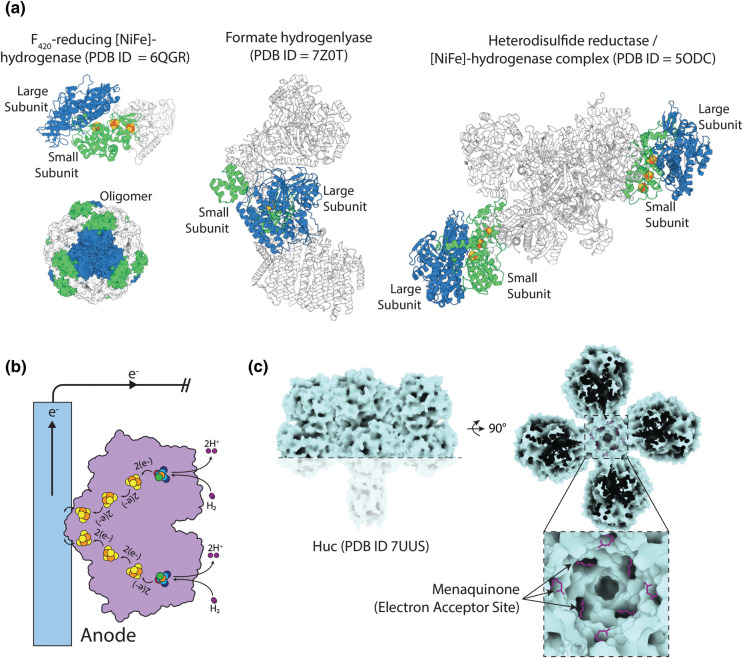
The complex structural of native [NiFe]-hydrogenases limits electron transfer to electrodes. (**a**) Structures of examples of [NiFe]-hydrogenases that form parts of larger multisubunit complexes. (**b**) A schematic showing the optimal orientation of a [NiFe]-hydrogenase, for electron transfer, when associated with an electrode. (**c**) A cutaway surface view of the Huc complex structure shows that the electron-acceptor sites, with menaquinone bound, are located in an internal chamber of the complex, which shields them from the bulk solvent.

[NiFe]-hydrogenases often form complexes composed of multiple subunits in addition to the large and small subunits of the core catalytic complex. These additional subunits are often responsible for delivering electrons to/from the enzyme substrates or coupling the hydrogenase to a larger redox-enzyme complex (Figures 1b, 4a) [9,40,92–94]. The generally preferred method of incorporation of [NiFe]-hydrogenases within the anodes of EBFCs is the direct attachment of the enzyme to the anode surface, orienting the terminal [FeS] cluster for efficient direct electron transfer (Figure 4b) [95]. Additional non-catalytic [NiFe]-hydrogenase subunits are likely to interfere with this process, lowering the efficiency of electron transfer. This is very likely the case for Huc, which consists of a large complex with an internal hydrophobic chamber that largely isolates the electron transfer sites (Figure 4c) [31,40]. For the use of [NiFe]-hydrogenases in EBFCs, it will be advantageous to rationalise them to the minimum structural unit required efficiently to perform H_2_ oxidation. Where hydrogenases are part of large complexes or contain membrane-associated components, such approaches will also likely assist in producing stable enzymes with higher yields. Attempts have been made to produce minimal active [NiFe]-hydrogenases, concluding that both the large and small catalytic subunits are required for enzyme activity and stability [96,97]. In the case of Huc, a minimal variant would need to maintain its key property of O_2_ insensitivity.

An additional challenge for the efficient coupling of [NiFe]-hydrogenases to the EBFC anode is that enzyme molecules are likely to adhere to the anode surface at random orientations, meaning many of the enzymes will not be appropriately orientated for efficient electron transfer, which will affect the power density of the fuel cell. This issue has been addressed by several groups by engineering the chemistry of both the hydrogenase and the electrode to maximise the percentage of enzymes that adhere to the electrode in the correct orientation [18,95,98–101]. For example, Rüdiger et al. crosslinked [NiFe]-hydrogenase from *D. gigas* to a 4-aminothiophenol-functionalised gold anode, positioning the distal [FeS] cluster proximal to the anode surface, leading to efficient electron transfer [100]. Work by Monsalve et al. [101] showed that an EBFC with an anode coated with positively charged carbon nanotubes and functionalised with membrane-bound hydrogenase from *A. aeolicus,* generated more than twice as much catalytic current density compared with negatively charged carbon nanotubes. Consistent with this finding, work by Xu et al. showed an EBFC containing a positively charged carbon paper anode coated with membrane-bound [NiFe] hydrogenase from *D. vulgaris* Miyazaki F performed significantly better than either a negatively charged or neutral equivalent. The authors attributed these findings to the presence of a negatively charged electron donor site on the enzyme, binding to the electrode via electrostatic interactions [18].

## Summary

As discussed above, significant work over the past two decades demonstrates that [NiFe]-hydrogenases are effective catalysts for the oxidation of H_2_ in EBFC. Hydrogenase-based EBFCs are currently inferior to PEMFCs with respect to turnover rates per volume, long-term stability, and coupling efficiency, though various innovative approaches have been taken to improve their performance. Where EBFCs show great promise is their catalytic selectivity and poison resistance compared with PEMFCs. The recent discovery of oxygen-insensitive hydrogenases such as Huc in particular opens paths to broaden the feedstock of hydrogen fuel cells to waste gas mixtures rather than purified hydrogen streams, with potential economic and sustainability benefits. Moreover, the exceptionally high affinity of these specialised [NiFe]-hydrogenases could allow EBFCs to produce energy from H_2_ in dilute gas mixtures or even from the air itself. Innovative enzymes, bioprocessing, and fuel cell engineering will be critical to optimise performance and enable the scalability of these technologies.

## Perspectives

- [NiFe]-hydrogenases have promise as fuel cell catalysts but this has been limited by their O_2_ sensitivity. However, the discovery of the O_2_-insensitive family member Huc has the potential to overcome this.
- [NiFe]-hydrogenases have a number of advantages over the platinum-based catalysts currently used in most H_2_ oxidising fuel cells.
- To realise this potential, further work is required to engineer [NiFe]-hydrogenases like Huc and the organisms that produce them to improve their activity, stability, and yield.

## Open Access

Open access for this article was enabled by the participation of Monash University in an all-inclusive *Read & Publish* agreement with Portland Press and the Biochemical Society under a transformative agreement with CAUL.

## Abbreviations

EBFCs: enzyme-based fuel cells
PEMFCs: proton-exchange membrane fuels cells
